# Genetic and immunological biomarkers predict metastatic disease recurrence in stage III colon cancer

**DOI:** 10.1186/s12885-018-4940-2

**Published:** 2018-10-19

**Authors:** Andreas Sperlich, Alexander Balmert, Dietrich Doll, Sabine Bauer, Fabian Franke, Gisela Keller, Dirk Wilhelm, Anna Mur, Michael Respondek, Helmut Friess, Ulrich Nitsche, Klaus-Peter Janssen

**Affiliations:** 1 Department of Surgery, Klinikum rechts der Isar, TUM, Ismaninger Str. 22, 81675 Munich, Germany; 2 Darmzentrum Vechta, St. Marienhospital, Marienstraße 6-8, 49377 Vechta, Germany; 3 Institute of Pathology, Technical University of Munich, Trogerstr. 18, 81675 Munich, Germany; 4 St. Marienhospital, Praxis für Pathologie Vechta, Marienstr. 11, 49377 Vechta, Germany

**Keywords:** Chemotherapy, Disease-free survival, Predictors of recurrence, KRAS, BRAF, SASH1, Microsatellite instability, Prognosis, Fluorouracil

## Abstract

**Background:**

Even though the post-operative outcome varies greatly among patients with nodal positive colon cancer (UICC stage III), personalized prediction of systemic disease recurrence is currently insufficient. We investigated in a retrospective setting whether genetic and immunological biomarkers can be applied for stratification of distant metastasis occurrence risk.

**Methods:**

Eighty four patients with complete resection (R0) of stage III colon cancer from two clinical centres were analysed for genetic biomarkers: microsatellite instability, oncogenic mutations in KRAS exon2 and BRAF exon15, expression of osteopontin and the metastasis-associated genes SASH1 and MACC1. Tumor-infiltrating CD3 and CD8 positive T-cells were quantified by immunocytochemistry. Results were correlated with outcome and response to 5-FU based adjuvant chemotherapy, using Cox’s proportional hazard models and integrative two-step cluster analysis.

**Results:**

Distant metastasis risk was significantly correlated with oncogenic KRAS mutations (*p* = 0.015), expression of SASH1 (*p* = 0.016), and the density of CD8-positive T-cells (*p* = 0.007) in Kaplan-Meier analysis. Upon multivariate Cox-regression analysis, KRAS mutation (*p* = 0.008) and density of CD8-positive TILs (*p* = 0.009) were retained as prognostic parameters for metachronous distant metastasis. Integrative two-step cluster analysis was used to combine all genetic markers, allowing stratification of patient subgroups. Post-operative distant metastasis risk ranged from 31% (low-risk) to 41% (intermediate), and 57% (high-risk) (*p* = 0.032). Increased expression of osteopontin (*p* = 0.019) and low density of CD8-positive T-cells (*p* = 0.043) were significantly associated with unfavourable response to 5-FU.

**Conclusions:**

Integrative biomarker analysis allows stratification of stage III colon cancer patients for the risk of metastatic disease recurrence and may indicate response to 5-FU. Thus, biomarker analysis might facilitate the use of adjuvant therapy for high risk patients.

**Electronic supplementary material:**

The online version of this article (10.1186/s12885-018-4940-2) contains supplementary material, which is available to authorized users.

## Background

Colorectal cancer is the third most common malignancy worldwide, regarding incidence and mortality [1]. Approximately 30% of patients with colorectal cancer present with local lymph node spread but no distant metastasis at the time of diagnosis (UICC/AJCC stage III). Tumor-specific five-year survival varies widely within this stage, ranging from 89% for stage IIIA to 36% for stage IIIC [2]. Therefore, therapy management is complex for patients with stage III disease, even though oncological tumor resection including lymph node dissection is still the condition precedent for cure. Since the early 1980s, 5-FU based adjuvant chemotherapy with or without Oxaliplatin was established for patients with stage III colorectal cancer [3–5]. Adjuvant chemotherapy in stage III colorectal cancer leads to a reduction of tumor recurrence to approximately 30%, from 50% without chemotherapy [6], yet simultaneously exposing all patients to considerably harmful side effects. Today, in the era of personalized therapy, the benefit of one standardized regimen of systemic chemotherapy for all stage III patients [5] has been challenged [7]. Neither the TNM classification system [2] nor currently available histopathological or biomarkers [8] warrant stratification for the prediction of recurrence risk in this stage.

In order to address this unmet clinical need, we and others have utilized biomarker-based approaches to specifically predict the risk of post-operative disease recurrence in the form of distant metastasis in colon cancer. This was successfully shown in stage II, but also partly in stage III CRC for molecular genetic markers, e.g., in the form of commercial kits or generic marker panels [9–16] as well as for protein-based assays [10, 17]. In addition to genetically defined markers, tumor-infiltrating T-cells have been proposed as crucial prognostic indicators. Importantly, the density of tumor-infiltrating T-lymphocytes (TIL) has stronger predictive power for patients’ survival than the well-established tumor-, node- and metastasis-classification system [18, 19]. In accordance, the gut microbiome and the intratumoral inflammatory cytokine profile are increasingly recognized to be associated with prognosis [20–25].

Of note, large-scale “omics” based approaches generally use an inductive marker selection without mandatory knowledge about biological function. In contrast, the present retrospective study was based on a deductive strategy. Genetic markers were selected to represent the pathways most frequently altered in colon cancer, such as the WNT-pathway (surrogate marker osteopontin), mutations in the oncogenes *KRAS* and *BRAF*, and the DNA microsatellite status. In addition, two genes that either suppress (*SASH1*) or induce (*MACC1*) metastasis in colon cancer were included. By combining this panel of genetically defined biomarkers that had previously been shown to be prognostic in stage II colon cancer [9], with the density of intratumoral T-cells (CD3/CD8), we aimed to predict the individual risk of metachronous metastasis, and response to 5-FU based adjuvant therapy in stage III colon cancer. Furthermore, we aimed to address the question whether stage II and stage III colon cancer can be stratified by similar biomarkers, or rather present different diseases altogether.

## Methods

### Tissue samples

Eighty four patients with stage III primary colon cancer were analysed, who underwent curative surgery (R0) at two centres and gave informed consent prior to surgery, from 1987 to 2014 (Table 1). Thorough testing ensured that there were no period effects during the accrual time period (Additional file 1: Figure S1). Eighty one patients were included from the Department of Surgery, TUM, Munich (TUM). The tissue was shock-frozen immediately after resection. Eight patients with stage III colon cancer were analysed from the academic teaching hospital St. Marienhospital Vechta, Germany (VECHTA). Due to excessive RNA degradation, five cases from Vechta had to be excluded. In addition, non-malignant colon mucosa from 79 patients with stage II or III disease, and colon cancer samples from 222 patients with stage II colon cancer were available for analysis from the predecessor study [9]. All tissue samples were stored in RNAlater solution (InVitrogen), or in DMEM cell culture medium supplemented with antibiotics/antimycotic immediately after surgery, and shipped to TUM over night for global analysis. Clinicopathological characteristics of the patients are summarized in Table 1. Patients with documented local recurrence were not included in this study in order to circumvent putative bias by surgical technique, but to warrant that only the intrinsic tumor biology is reflected in the systemic disease recurrence rate [10]. High ethical standard of this study was assured by supervision of the ethics committee of the Faculty of Medicine, TUM, which approved the study (# 1926/07), and by the ethics board of Marienhospital Vechta.

**Table 1:** Clinical characteristics of the patient collective

		All patients		Occurrence of metastasis		
		*n* = 84	(100%)	Metachr. Metastasis (*n* = 37)	Metachr. Metastasis (44%)	*p* (metachr. Met.)
Sex	Male	54	(64)	25	(46)	0.578
	Female	30	(36)	12	(40)	
Age (years, median)		67 (range 35–86)		37	(44)	0.428
Period of enrollment	before 1993	42	(50)	22	(52)	0.124
	1994 and after	42	(50)	15	(36)	
Sidedness	Right colon	35	(42)	17	(49)	0.633
	Left colon	44	(52)	19	(43)	
	Unknown	5	(6)	1	(20)	
Histology	Adenocarcinoma	75	(89)	34	(46)	0.076
	Mucinous adenocarcinoma	7	(9)	1	(14)	
	Signet ring cell	2	7 (2)	2	(100)	
Grading	Low (G1–2)	48	(57)	18	(38)	0.163
	High (G3–4)	36	(43)	19	(53)	
pT	T1	0	(0)	0	(0)	0.054
	T2	6	(7)	4	(67)	
	T3	57	(68)	20	(35)	
	T4	21	(25)	13	(62)	
pN	N1	58	(69)	19	(33)	0.002^a^
	N2	26	(31)	18	(69)	
Lymphatic invasion	L0	57	(68)	25	(44)	0.960
	L+	27	(32)	12	(44)	
Angioinvasion	V0	79	(94)	34	(43)	0.459
	V+	5	(6)	3	(60)	
Adjuvant chemotherapy	None	41	(49)	16	(39)	0.500
	5-FU alone	27	(32)	12	(44)	
	other	16	(19)	9	(56)	
Alive status	Alive	31	(37)	2	(6)	< 0.001
	Tumour rel. Death	33	(39)	33	(100)	
	Non-tumour rel. Death	20	(24)	2	(10)	
Occurrence of met.	No metastases	47	(56)	0	(0)	–
	Metachronous metastasis	37	(44)	37	(100)	

Note: The p-value refers to differences in the distribution of the factors regarding the risk of metachronous metastasis

^a^favouring pN1

### DNA and RNA extraction

20 to 30 mg of frozen tumor tissue was collected using a cryostat microtome (CM3050 S, Leica Microsystems, Wetzlar, Germany). Histology-guided sample selection [10] was performed by a pathologist to ensure a sufficient amount of tumor cells (> 30%). DNA and RNA were obtained using the Qiagen® AllPrep DNA/RNA Mini Kit (Qiagen GmbH, Hilden, Germany), according to the manufacturer’s protocol.

### Mutations in KRAS exon 2

The mutational status of the gene KRAS (codon 12 and 13 in exon 2) was analysed by high-resolution melting analysis of genomic DNA on a LightCycler® 480 II platform (Roche, Mannheim; SYBR Green I/HRM Dye Protocol), as described [9]. Analysis of genomic DNA from colon cancer cell lines with and without KRAS and BRAF mutations was performed in each run as control.

### Mutations in BRAF exon 15

The mutational status of the oncogene BRAF (V600E) was assessed by high-resolution melting analysis of genomic DNA on a LightCycler® 480 II platform (Roche, Mannheim), in a modification of published protocols [26]. 20 ng of genomic DNA (10 ng μl^− 1^) were amplified in total volume of 20 *μ*l with 10 *μ*l High-Resolution Master Mix, 2.4 mM MgCl_2_, and 0.25 mM each of oligonucleotide primers, 2 *μ*l template DNA and 5.2 *μ*l dH_2_O. Primer sequences were BRAF Exon 15 For: 5-GGT GAT TTT GGT CTA GCT ACA G-3, BRAF Exon 15 Rev.: 5-AGT AAC TCA GCA GCA TCT CAG G-3. After pre-incubation (95 °C, 10 min), amplification of a 147-bp product was carried out in 42 cycles (95 °C, 15 s/61 °C, 15 s/72 °C, 15 s), followed by melting point analysis with an initial phase: 95 °C, 5 s, and 72 °C, 90s, followed by a melting profile ranging from 72 °C to 95 °C in 19.2 min. As a positive control, genomic DNA from the BRAF-mutated colon cancer cell line HT29 was used.

### Microsatellite instability determination

Microsatellite instability (MSI) was tested with the MSI Analysis System, Version 1.2 (Promega, Mannheim, Germany). This assay co-amplifies the five mononucleotide repeat markers BAT-25, BAT-26, NR-21, NR-24, and MONO-27 to determine MSI status. Eighty one cases were analysed with the Bethesda panel (BAT25, BAT26, D2S123, D5S346, D17S250) using the Type-it Microsatellite PCR kit (Qiagen). MSI was defined when at least 2 of the 5 markers tested showed instability. The results of this assays have been demonstrated previously to be highly sensitive for MSI determination [27].

### Gene expression analysis

Extracted total RNA was assessed for degradation by denaturing gel electrophoresis and spectrometric measurement (ND-1000, Thermo Fisher Scientific, Wilmington, DE, USA), followed by quantification of rRNA 18S and 28S bands with the GelPlot macro in ImageJ software (NIH, Bethesda, MD, USA). The RNA of 84 patients (84% of all patients) was transcribed into complementary DNA (cDNA) and gene expression levels of Osteopontin, SASH1 and MACC1 were measured by quantitative real-time PCR, as described [9]. All gene expression levels refer to hypoxanthine-guanine phosphoribosyltransferase (HPRT) expression, relative to histologically confirmed normal colon mucosa pooled from 57 patients.

### Quantification of tumor-infiltrating lymphocytes

The quantification of CD3- and CD8-positive T-lymphocytes on tumor tissue sections has been carried out essentially as described [24, 25]. Briefly, tissue cryosections were fixed with 3% paraformaldehyde and stained with specific antibodies against CD3 (NeoMarker, 1:300), or against CD8 (BD Pharmingen, 1:300). Counterstaining was carried out with DAPI (Sigma-Aldrich, Munich, Germany), and secondary antibodies coupled to fluorophores were purchased from Jackson ImmunoResearch (West Grove, PA, USA). Sections were mounted in glycerol-gelatin (Sigma-Aldrich) and viewed using epifluorescence or confocal microscopes (Carl Zeiss, Jena, Germany). Slides were evaluated by two independent observers without knowledge of sample identity, based on a standardized surface area of 1.22 mm^2^ per tumor tissue. Images were composed and labeled using Adobe Photoshop Software (San Jose, CA, USA), and ImageJ (Scion Corporation, USA).

### Statistical analysis

Recurrence-free survival (i.e., distant metastasis-free survival) was considered as the primary endpoint for risk prediction. Statistical evaluation was performed using IBM SPSS Statistics software version 20.0 (SPSS Inc., Chicago, IL, USA). To derive optimal cut-off values of gene expression levels, maximally selected log-rank statistics performed by R Software version 2.13.0 (R Foundation for Statistical Computing, Vienna, Austria) were used. To consider multiple test issue within these analyses, the R-function ‘maxstat.test’ was employed [28]. Associations between protein expression and pathological features were given in crosstabs and were evaluated with chi^2^ test and Mann-Whitney-U test. Survival analysis was performed using Kaplan-Meier estimates. Cox’s proportional hazards regression analysis was used to investigate the effect on survival of multivariate relationships among covariates. Multivariate analysis was used for binary outcome data by logistic regression. Recurrence-free survival times as well as estimated hazard ratios were calculated and reported in 95% confidence intervals (CI). Clustering of the patients into different groups according to KRAS and BRAF mutation status, microsatellite stability status, and log-transformed gene expression levels of osteopontin, SASH1, and MACC1 was performed by the SPSS two-step cluster analysis function. Prognostic models that contain gene expression results were performed on the subset of 80 patients with all data available. All statistical tests were performed 2-sided, and the significance level was set at 0.05. No correction of *p*-values was applied to adjust for multiple test issue. However, results of all statistical tests being conducted were thoroughly reported so that an informal adjustment of *P* values can be performed while reviewing the data.

## Results

### Patient collective

Overall, 84 patients with complete resection (R0) of UICC stage III colon cancer were included from two independent clinical centres. Of note, no significant period effects were observed for clinical or molecular parameters (e.g., survival or frequency of genetic alterations) over the accrual period (Additional file 1: Figure S1). Rectal cancer was excluded, as this can be considered as distinct entity and prognosis would have been further influenced by neoadjuvant therapy [5]. Clinico-pathological data are shown in Table 1 (analysis by Chi-square test). A median of 19 lymph nodes was resected (range: 7 to 52). The median post-operative follow-up of the study group was 9.5 years. During follow-up, 37 patients (44%) developed distant metastasis after a median of 17 months, 33 (39%) patients died due to tumor-related causes after a median of 112 months. Five-year distant metastasis occurrence free-survival for the patient collective was 52 ± 6%. Among the clinical parameters, the nodal status (N-stage) was highly significantly associated with distant metastasis risk (*p* = 0.002), but the tumor-stage (T) barely attained significance (*p* = 0.054).

### Molecular alterations of metastasis-associated biomarkers

**Fig. 1:**
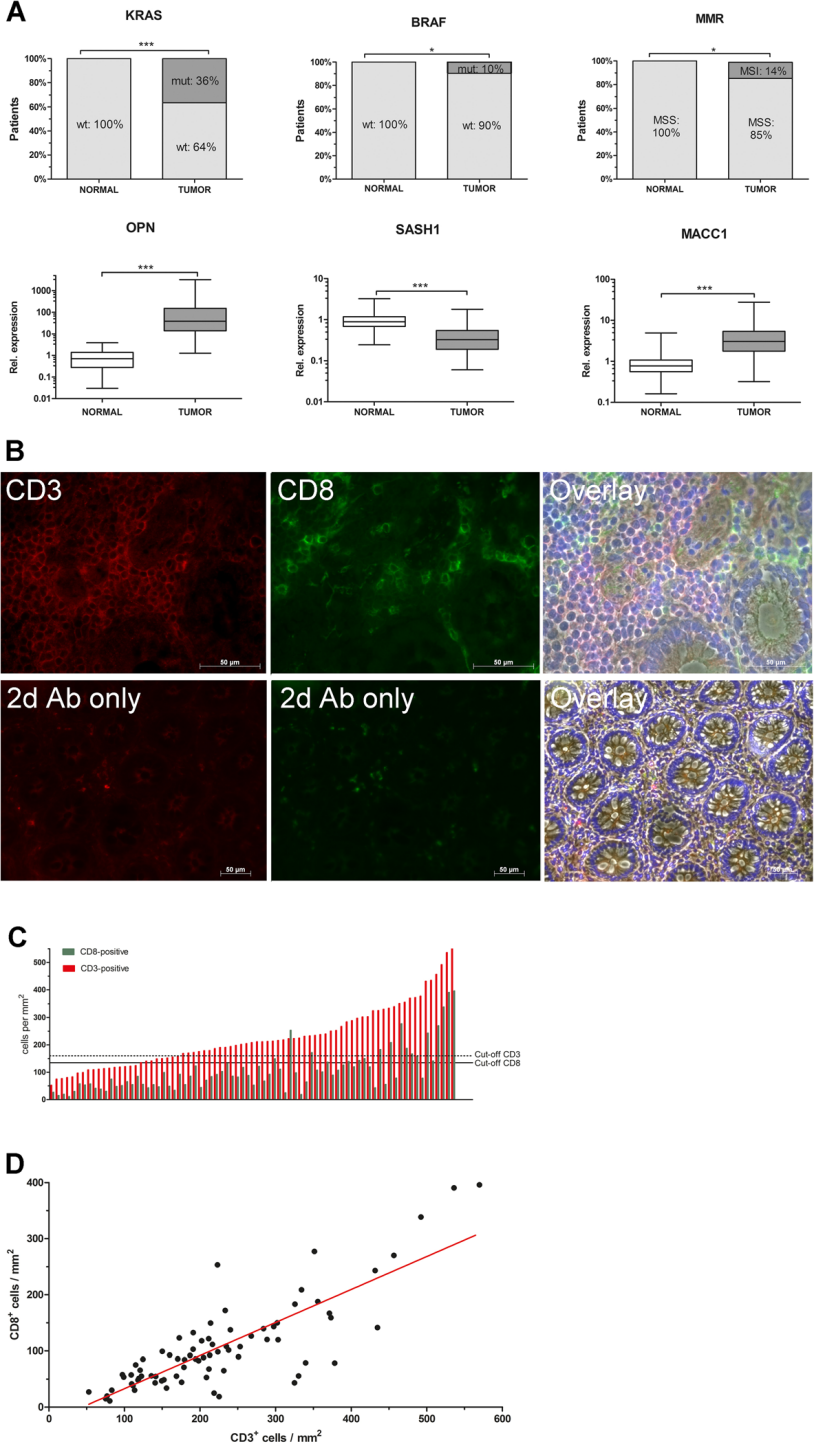
**a** Proportion of patients with oncogenic mutations in KRAS exon 2, mutations in BRAF exon 15, and microsatellite instable (MSI-H) tumors. Gene expression levels of osteopontin, SASH1, and MACC1. Matched non-diseased colon mucosa (*n* = 33 for KRAS-, BRAF- and MSI-analysis, and *n* = 79 for mRNA expression) and stage III colon cancer (*n* = 83). mut, mutated; MMR, mismatch repair; MSS, microsatellite stable; N, normal colon tissue; T, tumor; wt, wild type; analysis by t test. **b** Staining for Tumor-infiltrating T-cells (TILs) by immunocytochemistry on tissue sections. **c** Intratumoral densities of CD3 and CD8 T-cells, threshold for prognostic analysis. **d** Density of CD3 and CD8 TILs is highly significantly correlated (*p* < 0.0001; Spearman)

We tested a panel of six genetic biomarkers which were previously shown to predict metachronous distant metastasis in stage II colon cancer [9]. Oncogenic KRAS mutations (exon 2) occurred in 30 patients (36%), and BRAF mutations (exon 15) in 8 patients (10%), the mutations in both oncogenes were mutually exclusive. Non-diseased control tissue of 33 patients harbored no detectable KRAS or BRAF mutations (Fig. 1a). High-grade DNA microsatellite instability (MSI-High) was detected in 11 patients (13%). Microsatellite instable tumors occurred preferentially in patients with BRAF mutation (26% vs 2% in patients with BRAF wildtype, *p* < 0.001), and were located in the right colon in the majority of cases (90% vs. 10% in left tumors, *p* = 0.002, not shown). Female patients were more prone to microsatellite instability (24% vs 8% in male, *p* = 0.042) and BRAF mutation (20% vs 4% in male, *p* = 0.016). In accordance with literature findings, the prevalence of microsatellite instability and BRAF mutations was increased in elderly patients [29]. The age for MSI-H patients was 72 ± 13 years (mean ± SD), whereas it was 65 ± 11 years for MSS patients (*p* = 0.049, t test). The mean age for BRAF mutant patients was 74 ± 8 years, whereas it was 73 ± 8 years for BRAF wild type patients (*p* = 0.069, t test).

Intratumoral expression of osteopontin (Fig. 1a), a surrogate marker for aberrant activation of the Wnt-pathway, as well as of the tumor suppressor SASH1 and the metastasis-associated gene MACC1 was analyzed by qRT-PCR, and compared to normal colon mucosa. Expression of all three transcripts was highly significantly altered in tumors compared to normal mucosa, elevated in the case of osteopontin and MACC1, and reduced for SASH1. Expression of SASH1 was correlated with MACC1 (*p* = 0.039; t test), as well as with osteopontin (*p* = 0.012). Compared to tumors from stage II colorectal cancer (*n* = 222), microsatellite instability was significantly less frequent, osteopontin expression was significantly increased, and SASH1 expression significantly decreased (Additional file 1: Figure S2).

### Quantification of tumor-infiltrating T-lymphocytes

Tumor-infiltrating T-lymphocytes (TILs) were identified by specific antibodies for the cell surface markers CD3 and CD8 by immunocytochemistry (Fig. 1b), and quantified by two independent observers blinded to the identity of the samples. We observed in tumors a median of 208 CD3-positive cells per mm^2^ (range: 53–570), and a median of 86 CD8-positive cells per mm^2^ (range: 11–396) (Fig. 1c). Of note, CD3 and CD8 densities in individual tumor samples were highly significantly correlated (*p* < 0.0001; Fig. 1d; t test). Further, the density of CD3-, as well as of CD4-positive TILs was negatively associated with expression of osteopontin (Table 2; Spearman test).

**Table 2:** Correlation of biomarkers

		KRAS	BRAF	MSI	OPN	SASH1	MACC1	CD3
BRAF	c.coeff.	**−0.243**						
	p	**0.034**						
MSI-H	c.coeff.	−0.197	**0.646**					
	p	0.098	**< 0.001**					
OPN	c.coeff.	0.021	0.177	0.208				
	p	0.858	0.127	0.08				
SASH1	c.coeff.	−0.115	0.012	− 0.007	**0.288**			
	p	0.324	0.921	0.956	**0.012**			
MACC1	c.coeff.	0.127	−0.118	−0.163	−0.013	**0.237**		
	p	0.272	0.311	0.171	0.909	**0.039**		
CD3	c.coeff.	−0.002	0.179	0.08	**−0.277**	−0.203	− 0.047	
	p	0.989	0.121	0.504	**0.015**	0.078	0.69	
CD8	c.coeff.	0.072	0.198	0.177	**−0.244**	−0.078	−0.044	**0.749**
	p	0.536	0.086	0.137	**0.034**	0.505	0.709	**< 0.001**

Correlation of biomarkers: The upper number in each box indicates the correlation coefficient (Spearman ρ). The lower number depicts the corresponding *P* value. Significant correlations in bold print

### Genetic and immunological biomarkers for prognosis and survival

**Fig. 2:**
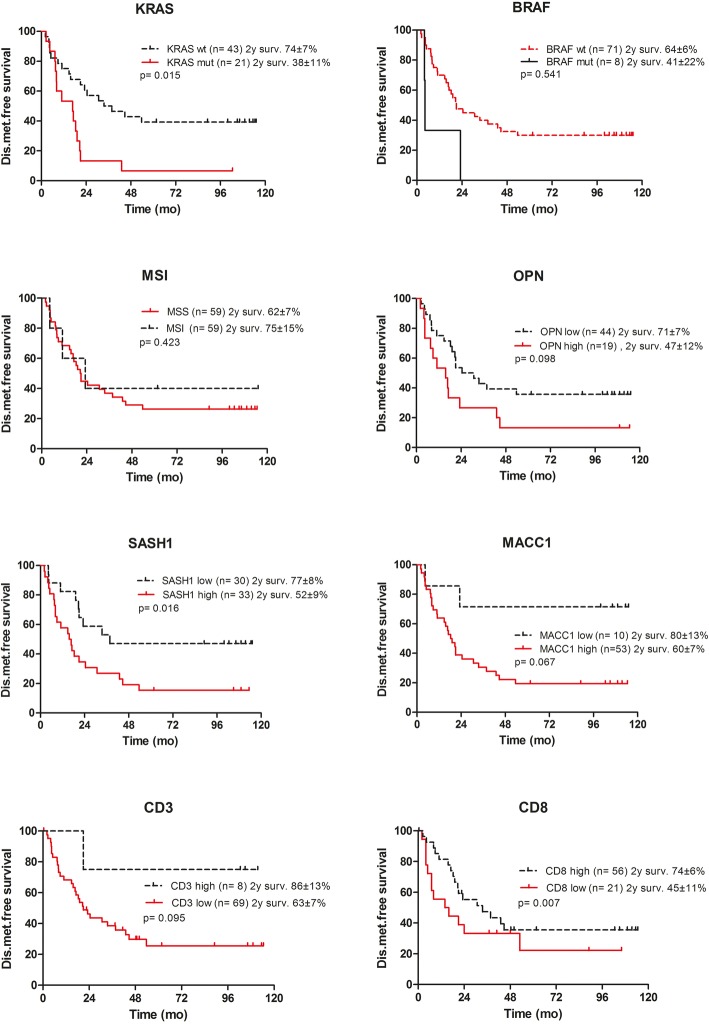
Distant-metastasis-free survival depending on the mutational status of KRAS, BRAF, microsatellite instability status, and of expression levels of Osteopontin, SASH1, MACC1, and of the density of CD3- and CD8-positive TILs, respectively. Kaplan-Meier *p*-values refer to log rank (Mantel Cox) statistics. OPN, Osteopontin; MSI-H, microsatellite instable high; MSS, microsatellite stable; mut, mutated; wt, wild type; 5 yr. surv, 5 year survival

As stated earlier, the nodal status (pN2 versus pN1) was the only clinico-pathological factor significantly associated with metachronous distant metastasis (Table 1). The time-dependent metastasis risk for patients with KRAS exon 2 mutations was significantly increased in Kaplan-Meier analysis (*p* = 0.015, log rank, Fig. 2). The BRAF exon 15 mutation status (*p* = 0.541, log-rank), as well as the MSI status (*p* = 0.423, log-rank) were not significantly associated with the risk of metastasis, possibly due to the relatively low frequency of events (BRAF mutations in *n* = 8, MSI-high status in *n* = 11 cases). Increased expression of osteopontin or MACC1 was associated with inferior prognosis, but failed to attain significance. Interestingly, an association between increased expression of SASH1 and reduced distant metastasis-free survival was found (*p* = 0.016). High density of TILs was associated with increased survival, highly significant in the case of CD8-positive lymphocytes (*p* = 0.007) (Fig. 2). Upon multivariate analysis, KRAS mutations (*p* = 0.008), as well as low density of CD8-positive TILs (*p* = 0.009) were retained as independent prognostic factors predicting metachronous distant metastasis-free survival (Table 3; multivariate Cox-regression analysis). Step-by-step inclusion of clinical and histopathological factors did not lead to relevant changes in the multivariate risk assessment (not shown).

**Table 3:** Multivariate Cox-regression analysis of the prognostic impact on metachronous distant metastasis

	*p*	HR	95% CI	
			Lower	Upper
KRAS (mutated)	**0.008**	3.21	1.36	7.59
BRAF (mutated)	0.106	5.45	0.67	42.42
MMR (MSI-H)	0.777	0.81	0.19	3.50
Osteopontin (continuously)	0.852	1.00	0.99	1.00
SASH1 (continuously)	0.103	3.01	0.80	11.31
MACC1 (continuously)	0.980	0.99	0.90	1.11
CD3 (continuously)	0.109	1.01	0.99	1.01
CD8 (continuously)	**0.009**	0.99	0.97	0.99

*CI* confidence interval, *HR* hazard ratio, *MMR* mismatch repair, *MSI-H* high-grade microsatellite instability

### Molecular subgroups with distinctive risk profiles

**Fig. 3:**
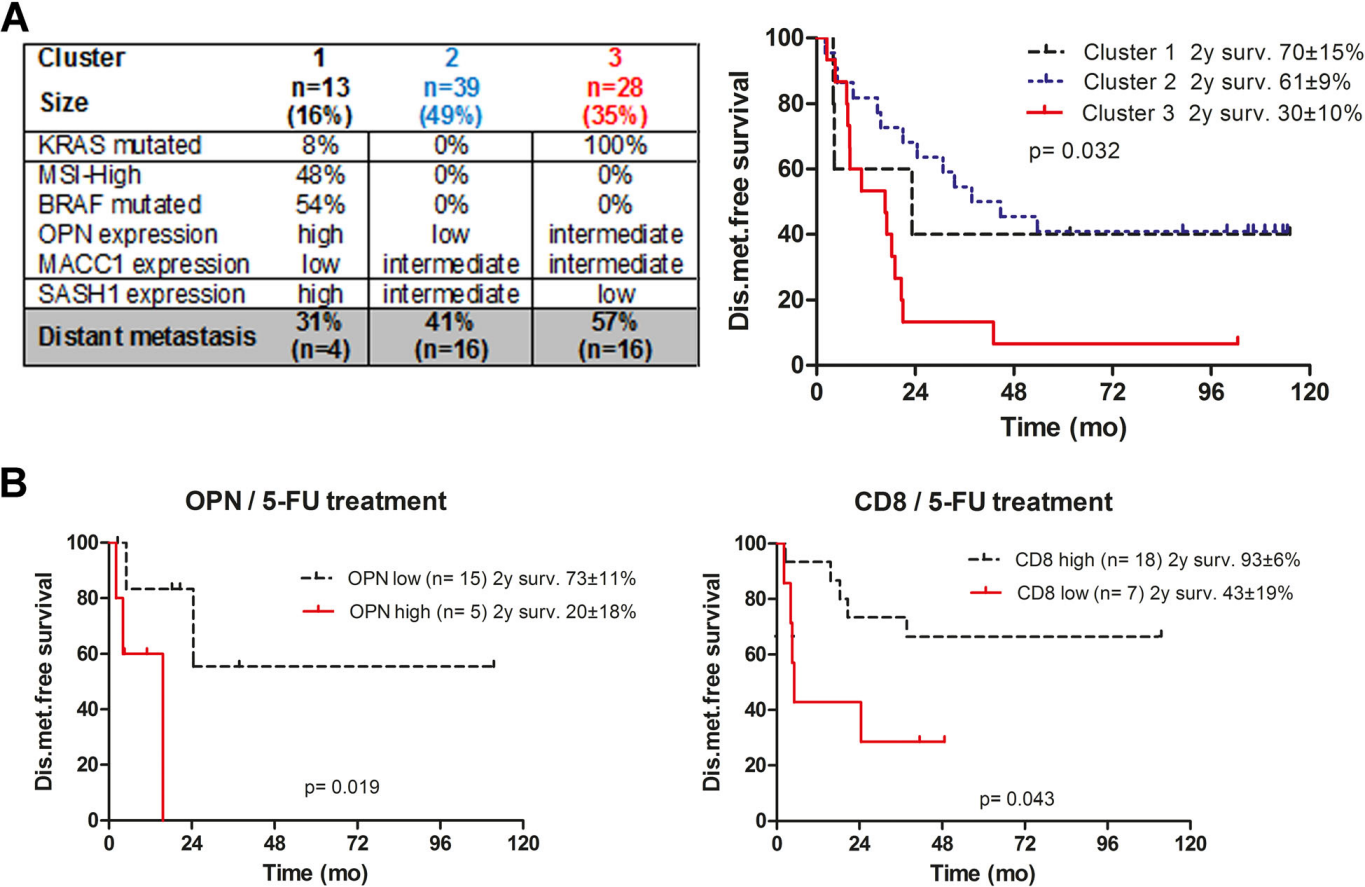
**a** Results of the unsupervised two-Step Cluster Analysis. Three groups of patients were identified depending on their molecular signature. The descending order of the molecular markers reflects the assumed significance of the predictor. Right panel: Distant-metastasis-free survival depending on the cluster allocation. Mut, mutation, MSI, high-grade microsatellite instability (Cluster analysis by R algorithm, log rank Mantel Cox). **b** In the subgroup of patients treated with 5-FU adjuvant monotherapy, distant-metastasis-free survival is associated with the intratumoral expression level of the WNT-marker osteopontin, as well as with intratumoral CD8 T-cell density (log rank)

**Fig. 4:**
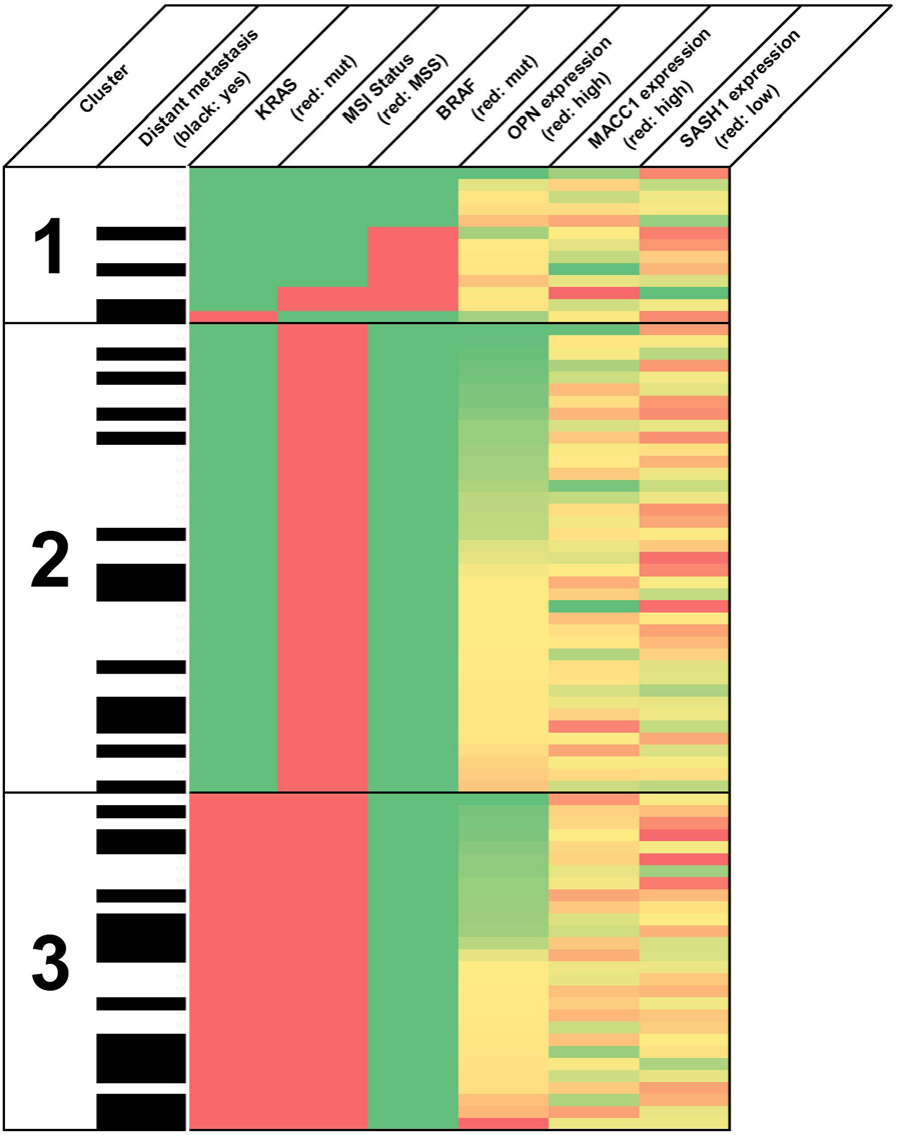
Graphical representation of the two-step cluster analysis, depicting the risk dependent clusters #1, #2, and #3. Every row represents one patient with his or her specific molecular genetic tumor profile. The occurrence of distant metastasis is indicated by filled bars, open bars indicate absence of distant metastasis. Prognostically poor specifications of the nominal variables are indicated as red bars (KRAS exon 2 mutation, BRAF exon 15 mutation, stable microsatellite status or MSS). For the continuous mRNA expression variables, the colour gradient from green to red indicates increasing biological aggressiveness (high levels of OPN and MACC1, low levels of SASH1)

In our previously published study on stage II CRC, we found that an unsupervised two-step cluster analysis of molecular biomarkers was superior to standard clinical TNM staging regarding the prediction of distant metastasis-free survival [9]. Here, we tested whether the same biomarker panel was clinically useful in nodal positive stage III patients, and compared the data with the previously published stage II dataset. Cluster-analysis automatically determines the number of pre-existing clusters and allows for integration of both continuous and categorical variables (Fig. 3a; graphical representation in Fig. 4; cluster analysis by R algorithm, log rank). According to their molecular genetic profile, the cluster analysis identified three distinct patient cohorts, with significantly varying risk of disease recurrence and 2 year distant metastasis-free survival ranging from 31% (cluster 1) to 57% (cluster 3, *p* = 0.032, log rank). There were no significant differences between the three clusters regarding patient sex or age. The low-risk cluster #1 was characterized by frequent BRAF mutations, DNA microsatellite instability, high expression of the Wnt-pathway surrogate marker osteopontin, low expression of the metastasis marker MACC1 and high expression of the tumor suppressor SASH1. Patients from the high-risk group (cluster #3) featured oncogenic KRAS mutation, stable DNA microsatellites, intermediate levels of osteopontin, high MACC1 expression, and reduced SASH1 levels. Patients from the intermediate risk group (cluster #2, 41% risk) showed neither a KRAS mutation nor BRAF mutation, had stable microsatellites, low osteopontin expression, and intermediate expression of both MACC1 and SASH1. However, inclusion of TIL densities (CD3/CD8) together with the molecular genetic biomarkers did not increase their prognostic power in two-step cluster analysis (Additional file 1: Figure S3).

### Biomarkers associated with response to adjuvant treatment

Biomarkers in stage III CRC are not only awaited for prognosis, but also for prediction of therapy response. Routinely used adjuvant chemotherapeutic regimes in stage III colon cancer often rely on combinations of drugs, changing frequently over the past years. Therefore, analysis was restricted to the subgroup of patients receiving 5-FU monotherapy (*n* = 25), which represents an oncological standard of care for decades, to ensure a more reliable basis for analysis. However, due to the relatively small size of this patient subgroup the statistical analysis has limited power. Among the clinical, molecular and immunological markers tested, only two were found to be associated with response to chemotherapy. Patients with high intratumoral expression of osteopontin (*p* = 0.019; t test), or low densities of CD8-positive TILs (*p* = 0.043) had significantly worse metastasis-free survival after adjuvant therapy (Fig. 3b). None of the other molecular or immunological parameters tested showed a significant association with response to adjuvant therapy (not shown).

## Discussion

Roughly one out of three patients first diagnosed with colorectal cancer presents with tumor dissemination to the local lymph nodes, but no distant metastasis (UICC/AJCC stage III). Importantly, there are strong interpatient variations regarding the prognosis and response to therapy, reflected in a range of tumor-specific five-year survival from 89% to only 36% [2]. Therefore, reliable biomarkers are urgently awaited for evidence-based clinical therapy management. We have tested here whether molecular or immunological biomarkers allow stratification of stage III patients for the risk of distant metastasis. We applied a biomarker panel that was successfully established on stage II colon cancer in a previous study [9]. However, it is currently unclear to which extent stage II and stage III of colon cancer differ on the molecular level, therefore we aimed to compare biomarkers for both cancer stages. The WNT-surrogate marker osteopontin was significantly increased in stage III tumors, compared to the published data from stage II tumors [9], in accordance with earlier findings demonstrating an association of osteopontin with tumor progression [30]. In contrast, the tumor suppressor SASH1 was highly significantly decreased in stage III compared to stage II tumors, again in accordance with previous findings [31]. The relative frequency of DNA mismatch repair defects, oncogenic mutations and overall survival rates in the cohort tested here are in good accordance with reported data, confirming the comparability with the general patient population [9, 32]. Upon individual analysis of the biomarkers by Kaplan-Meier analysis, prognostic significance was observed for oncogenic KRAS mutations, expression of SASH1, and low density of CD8-positive TILs. The positive prognostic effect of intratumoral CD8-positive TILs is in excellent accordance with earlier studies [19, 24, 33], as well the negative prognostic effects of oncogenic KRAS and BRAF mutations as reported from the PETACC-8 trial, even though our study does not allow to further distinguish codon 12/13 mutations in KRAS [34, 35].

Interestingly, a patient subgroup with relatively high intratumoral SASH1 expression showed increased distant metastasis occurrence risk in our study, as opposed to previous results that showed a negative prognostic effect for decreased or absent SASH1 expression [9, 31]. Intriguingly, a recent study in breast cancer reported a very similar observation. Even though SASH1 was globally down-regulated among all tumors and its expression associated with favorable prognosis, certain subgroups like estrogen receptor positive cancers showed a significantly reduced survival for cases with high SASH1 expression [36]. Therefore, the molecular context and tumor stage may significantly contribute to the biology of SASH1, which may extend beyond tumor suppression. Patients with increased MACC1 expression clearly showed decreased disease recurrence-free survival, even though this effect did not attain significance [37, 38].

Next, we used an unsupervised two-step clustering algorithm to described earlier in detail [9], including all tested biomarkers. Briefly, this statistical method allows the handling of both categorical (mutated vs. wild-type KRAS, BRAF, and MSI-staus) and continuous (expression values of OPN, SASH1, and MACC1, T-cell densities) variables. The algorithm automatically determines the optimal number of clusters, defining three patient groups with different risk of distant metachronous metastasis. Of note, the same algorithm previously defined four groups for nodal-negative stage II disease [9]. The three clusters identified here are in good accordance with the recent international consortium consensus classification for colorectal cancer, with subtypes CMS1 – CMS4 [32](Fig. 4). These subtypes comprise the following groups: CMS1 or “MSI immune” features microsatellite instability, BRAF mutations and high immune infiltration. CMS2 represents the “canonical” type with aberrant activation of the canonical WNT pathay. CMS3 has been called the “metabolic” subtype, with mixed MSI status, KRAS mutations and frequently deregulated cellular metabolism. Finally, CMS4 (“Mesenchymal”) shows strong stromal infiltration and angiogenesis, and shows the worst survival rates [32].Thus, the low-risk cluster #1 in the present study represents the MSI-high and BRAF mutated consensus molecular subtype 1 (CMS1 or “MSI immune”), with a prevalence of 16% in our cohort, and a 2-year survival rate of only 30%. Cluster #2 was the largest subgroup, comprising 49% of patients with a two-year survival of 61%. This group most likely represents the “canonical” consensus subtype (CMS2), characterized by somatic copy number alterations, no obvious mutations of the genes KRAS or BRAF, or instable microsatellites. Cluster #3, the high-risk group in our cohort with a two-year survival rate of 70%, is likely constituted of two consensus molecular subtypes, the “metabolic” CMS3 characterized by KRAS mutation, and the “mesenchymal” CMS4. 35% of patients are allocated to cluster #2. Thus, by a combination of simple and straightforward genetic tests, a clinically useful risk stratification for post-operative metastatic occurrence could be achieved. In clinical practice, our data allow the conclusion that high-risk patients (cluster #1) may be candidates for aggressive multimodal therapy. In contrast, low-risk patients might be spared the toxic side-effects of unnecessary chemotherapy, in line with recent discussions, even though their risk of distant metastasis remains clinically significant [7]. However, the putative benefits of an intensified therapy regime for high-risk patients, or reduced chemotherapy for the low-risk group, needs to be demonstrated independently in a prospective setting.

However, it is currently unclear whether the molecular and immunological markers tested here are able to predict the response to specific multimodal therapies, such as 5-FU based chemotherapy. Therefore, we analysed the predictive capacity of the biomarkers with respect to response to adjuvant therapy. We focused on 5-FU, as a standard component of chemotherapeutic regimes for several decades. The subgroup of patients receiving 5-FU monotherapy had significantly worse prognosis in case of increased osteopontin expression, or in case of low intratumoral densities of CD8-positive TILs. However, due to the relatively small size of this patient subgroup, these results have to be repeated independently before sound conclusions can be drawn. A reduced infiltration with cytotoxic T-cells may indicate a defective adaptive anti-tumoral immune response, which is likely required to achieve the full benefit of cytotoxic therapy. We previously identified osteopontin as negative prognostic marker, and a hallmark biomarker for aberrant activation of the canonical Wnt pathway [30, 39]. In fact, colorectal cancer cells were shown to upregulate Wnt signalling as an escape mechanism to 5-FU treatment [40]. Interestingly, the high-risk subgroup (Cluster #1) identified by unsupervised cluster analysis comprises the cases with high intratumoral osteopontin expression. Thus, even though patients from the high-risk cluster #3 would require aggressive multimodal therapy, in addition to surgical tumor resection, patients from cluster #3 may actually have an especially poor response to 5-FU. Biomarker analysis may allow to individualize the therapy regime, since recent data indicate that MSI-high patients with stage III disease specifically benefit from oxaliplatin, in contrast to 5-FU [41]. Further prospective studies are necessary to establish whether alternative or intensified cytotoxic drugs would indeed be more effective for high-risk subgroup.

## Conclusion

Personalized risk prediction for patients with lymph-node positive colorectal cancer (UICC/AJCC stage III) is currently not feasible based on clinical and pathological markers. We have shown here that a combination of established molecular and immunological markers allow stratification for the risk of post-operative distant metastasis. Taken together, integrative biomarker analysis holds the potential to facilitate personalized therapy, helping to reduce the rate of distant metastatic tumor occurrence in the high-risk group, as well as putative side effects of unnecessary chemotherapy in the low-risk group.

## Additional file

Additional file 1: Data in support. (PDF 387 kb)
