# Supplementary material for: Genetic and immunological biomarkers predict metastatic disease recurrence in stage III colon cancer
Source: BMC Cancer. 2018 Oct 19;18:998. doi: 10.1186/s12885-018-4940-2 (PMC6194664; doi:10.1186/s12885-018-4940-2)
Supplement: Supplementary file 1 — Data in support. (PDF 387 kb) [file 12885_2018_4940_MOESM1_ESM.pdf]

Supplementary Figure 1

A

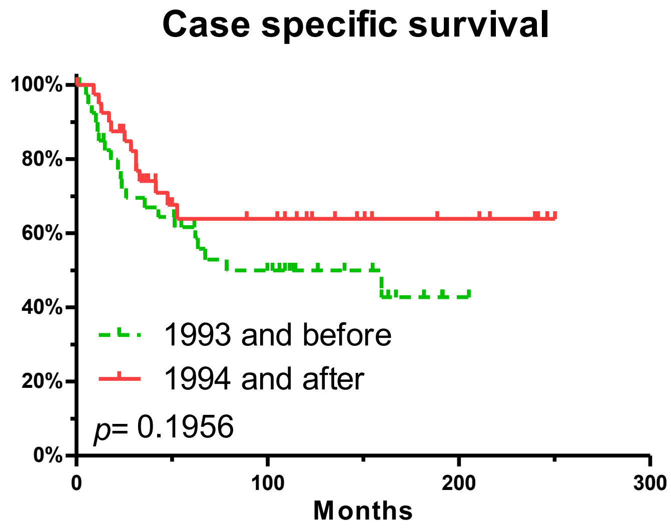

B

Biomarker	1993 and before	1994 and after	p-value
KRAS mutated	29%	44%	0,174
MSI-High	17%	11%	0,525
BRAF mutated	12%	7%	0,713
OPN expression high	31%	33%	0,999
MACC1 expression high	36%	35%	0,999
SASH1 expression high	48%	35%	0,270

Supplementary Figure 1: Analysis of period effects during the accrual time of the study. (**A**) Differences in case-specific survival were tested by Kaplan Meier analysis for patients included earlier, or later than the median (year 1993). (**B**) No significant differences were observed for any of the tested molecular parameters, when comparing patient tissue before or after the median (1993).

Supplementary Figure 2

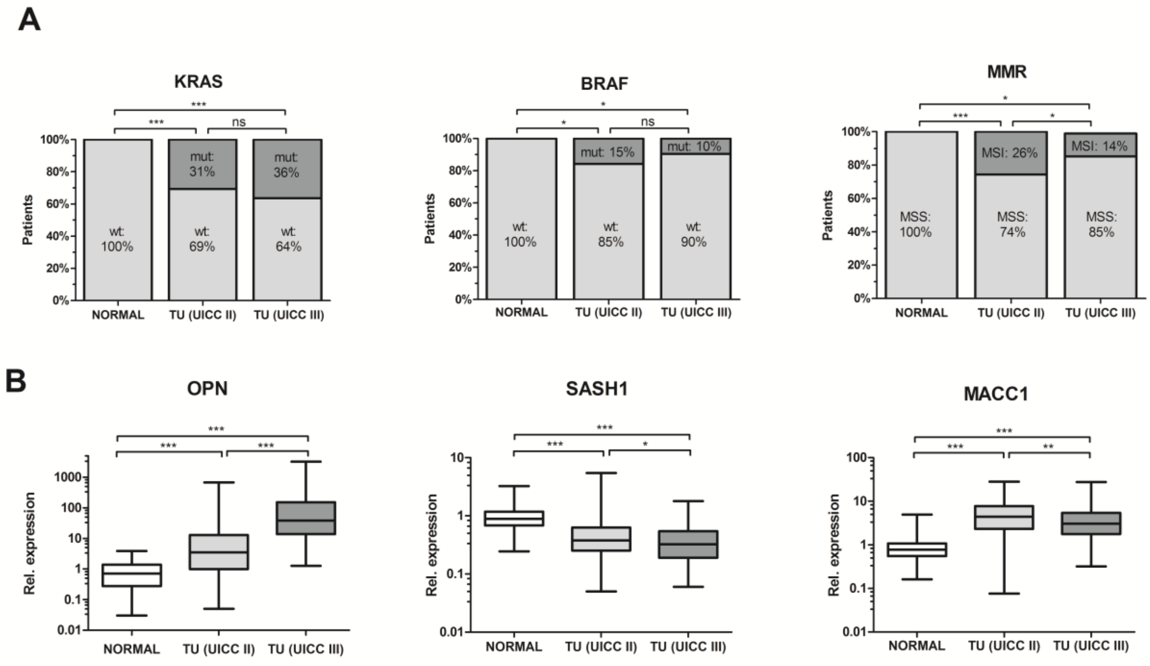

Supplementary Figure 2: Proportion of patients with (**A**) mutations in KRAS exon 2, mutations in BRAF exon 15, and microsatellite instable (MSI-H) tumors. The graphs show normal, non-diseased colon mucosa (n=33), tumor samples from UICC stage II colon cancer (n=222), and stage III colon cancer (n=83). (**B**) Gene expression levels of Osteopontin, SASH1, and MACC1. The graphs show normal, non-diseased colon mucosa (n=79), tumors samples from UICC stage II colon cancer (n=172), and stage III colon cancer (n=82). mut, mutated; MMR, mismatch repair deficiency; MSI: high-grade microsatellite instability; MSS, microsatellite stable; NORMAL, normal colon tissue; TU, tumors; wt, wild type. Mann-Whitney test, ns, not significant, *p<0.05; **p<0.01;***p<0.001.

Supplementary Figure 3

Patient allocation and prediction of prognosis by two-Step Cluster Analysis (including CD3/CD8+ TILs)

**Cluster**<br>**Size**	**1**<br>**n=40**<br>**(56%)**	**2**<br>**n=26**<br>**(36%)**	**3**<br>**n=6**<br>**(8%)**
BRAF mut	0%	0%	100%
KRAS mut	0%	100%	0%
MSI	8%	4%	17%
CD8 expression	low	intermediate	high
CD3 expression	low	intermediate	high
OPN expression	low	intermediate	high
MACC1 expression	intermediate	high	low
SASH1 expression	high	low	intermediate
**Distant metastasis**	**33% (n=13)**	**58% (n=15)**	**33% (n=2)**

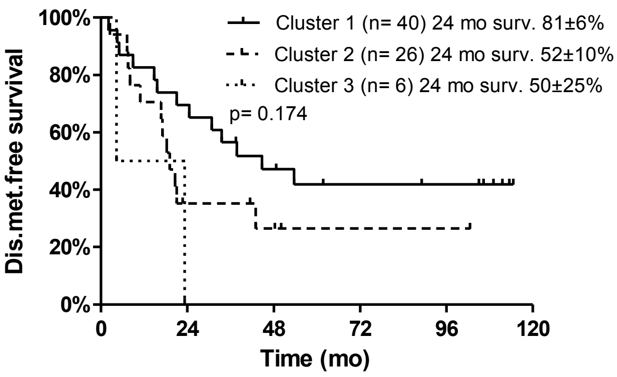

Supplementary Figure 3: Left panel: results of unsupervised two-Step Cluster analysis, including molecular and immunological biomarkers. Three groups of patients were identified depending on their molecular signature and relative risk of metachronous distant metastasis. The descending order of the biomarkers reflects the assumed significance of the predictor. Right panel: time-dependent distant-metastasis-free survival depending on the cluster allocation shows no significant differences. mut, mutation, MSI, high-grade microsatellite instability.
